# Privacy-Preserving Energy-Aware Routing for Partially Sustainable IoT Networks in Smart Cities

**DOI:** 10.3390/s26217015

**Published:** 2026-11-06

**Authors:** Ibrahim Mutambik

**Affiliations:** Department of Computer Science and Engineering, College of Applied Studies, King Saud University, Riyadh P.O. Box 11451, Saudi Arabia; imutambik@ksu.edu.sa

**Keywords:** smart cities, partially sustainable IoT networks, energy data privacy, differential privacy, energy-depletion attacks

## Abstract

Smart-city wireless sensor and Internet of Things (IoT) networks are progressively transitioning toward sustainable energy sources. During this transition, rechargeable energy-harvesting nodes must coexist with conventional devices powered by non-rechargeable batteries, creating partially sustainable IoT networks with conflicting energy-management objectives. Energy-harvesting nodes seek energy-neutral operation, whereas conventional nodes must minimize consumption to extend their operational lifetime. Coordinating these devices requires the exchange of energy information (EI), particularly battery state of charge. However, disclosing such information can enable adversaries to identify energy-constrained nodes and target them through energy-depletion attacks. This study examines the existing Dual Energy PROfile for interNet-of-thiNgs with Enhanced Security (DEPRONN-ES) framework for heterogeneous smart-city IoT networks. The framework integrates dual-profile energy management, energy-aware routing, adaptive data aggregation, and differential privacy. The focus is their coordinated application to heterogeneous energy resources and privacy-preserving EI exchange, rather than the introduction of new individual techniques. The framework was implemented using Contiki-NG and evaluated through Renode emulation with real indoor light-intensity traces from Columbia University to model energy harvesting by rechargeable nodes. The results show that, with a privacy budget of 0.1, DEPRONN-ES reduces the attacker’s target-identification success rate from 100% to 13.33%, representing an 86.67-percentage-point reduction. This reduction concerns targeting accuracy and does not imply prevention of malicious traffic. It also enables sustainable and conventional nodes to operate according to their distinct energy objectives. The reported findings suggest potential benefits from coordinating privacy protection and energy management under the evaluated conditions.

## 1. Introduction

Billions of connected sensors and embedded devices now form extensive Internet of Things (IoT) ecosystems that continuously generate, exchange, and analyze data. Within smart cities, this infrastructure supports diverse services, including intelligent transportation, environmental surveillance, utility management, residential applications, and industrial operations [1,2], but the increasing scale of these deployments has intensified concerns about power demand, finite resource use, and electronic waste. This strengthens the need for energy-sustainable IoT solutions and environmentally responsible urban technologies [3,4].

Although individual IoT devices are typically compact and energy-efficient, their collective environmental footprint can become considerable on an urban scale. Trackers, smart meters, and wireless sensors operate using single-use batteries that require periodic replacement. The resulting accumulation of discarded devices and power cells adds to electronic waste, an impact that may be overlooked because each node consumes only a small amount of energy, plus some conventional battery chemistries contain toxic metals, such as mercury, cadmium, or lead, which may enter terrestrial and aquatic ecosystems if disposal is not properly managed [5,6]. Limiting battery replacements and prolonging device operation are therefore important for both network efficiency and the environmental sustainability of city-wide sensing systems.

**Figure 1:**
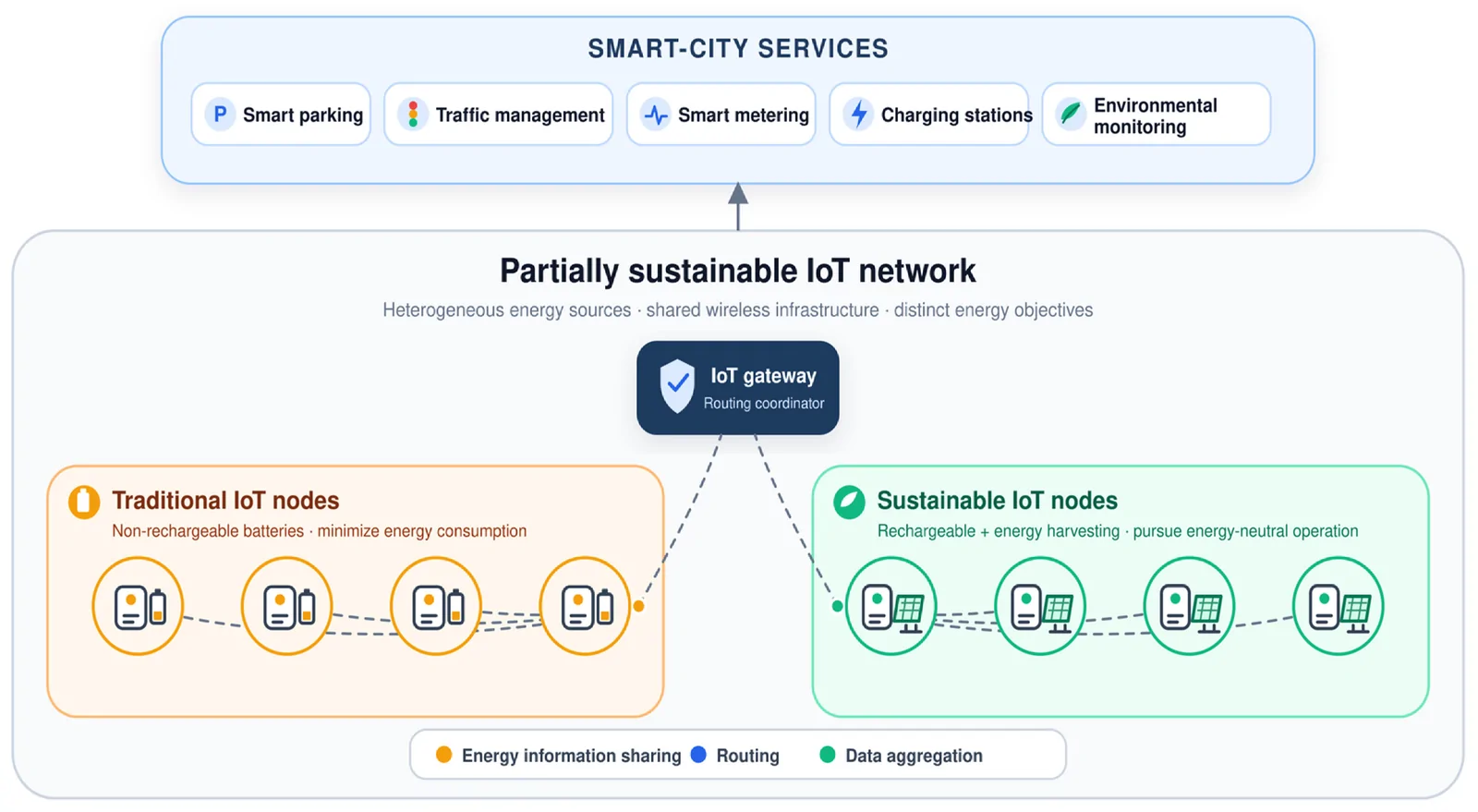
Coexistence of conventional battery-powered and sustainable energy-harvesting IoT nodes in a partially sustainable smart-city network.

One pathway toward greener IoT networks is the use of rechargeable batteries combined with energy-harvesting technologies that capture solar, kinetic, thermal, or radio-frequency energy [7,8]. However, replacing all existing IoT devices with energy-harvesting alternatives is neither economically nor technically feasible in the short term. Many legacy devices remain dependent on non-rechargeable batteries because of their hardware specifications, deployment constraints, replacement costs, or incompatibility with renewable-energy components. Rechargeable energy-harvesting nodes must coexist with conventional battery-powered devices, creating partially sustainable IoT networks [9,10], and as city-scale infrastructures are replaced incrementally, the transition from conventional to fully sustainable IoT networks will be gradual [11,12]. Figure 1 illustrates the services that such heterogeneous networks may support.

The two device categories have different energy-management requirements. Nodes powered by finite, non-replenishable batteries need to conserve their available charge, whereas energy-harvesting devices can use renewable energy and follow an energy-neutral strategy [13,14]. A common control policy can therefore be inefficient as it may leave harvested energy unused or assign excessive communication workloads to conventional devices with limited reserves. Partially sustainable deployments require energy-aware differentiation among node types when routes are established, relay duties are allocated, and aggregation ratios are selected.

Energy-aware coordination relies on the exchange of energy information (EI), including battery type, residual energy, and state of charge. Wireless IoT nodes use this information to identify suitable forwarding paths, avoid energy-constrained devices, and distribute network traffic more efficiently [15], but precise EI can also reveal which nodes are most vulnerable, and an adversary with access to this information can direct excessive traffic towards them. This shortens network lifetime, potentially disrupting essential smart-city services [16,17].

Protecting EI in resource-constrained IoT networks remains challenging. Cryptographic mechanisms can protect information during transmission, but even lightweight schemes may introduce computational, memory, and communication overhead that is significant for constrained devices [18,19]. Energy-aware routing solutions may also exchange battery information in plaintext, allowing malicious nodes to identify and target devices with the lowest available energy [20,21]. Consequently, an effective solution must retain sufficient EI for routing and aggregation decisions while limiting the exposure of precise battery conditions.

Energy-aware routing, data aggregation, differential privacy, RPL, and energy harvesting are established techniques. Their coordination nevertheless raises a specific design challenge: routing and aggregation depend on energy information whose disclosure can assist an adversary. Privacy perturbation may reduce that disclosure, but it can also affect the accuracy of network-control decisions. These interactions must be considered alongside the different energy-management objectives of conventional and energy-harvesting nodes.

The existing Dual Energy PROfile for interNet-of-thiNgs with Enhanced Security (DEPRONN-ES) framework addresses this combined problem through dual energy profiling, energy-aware routing, adaptive aggregation, and privacy-preserving EI exchange. The present study examines this framework and the interaction of its constituent mechanisms. It does not claim the invention of RPL, energy harvesting, data aggregation, differential privacy, or their original integration in DEPRONN-ES.

Its Dual Energy Profile Mechanism (DEPM) is embedded in a tree-structured routing topology and uses device-specific power conditions to choose upstream forwarding nodes and set appropriate aggregation ratios. This coordination is intended to conserve non-replenishable energy while allowing harvesting-enabled devices to use replenished resources. For privacy protection, DEPRONN-ES perturbs reported numerical EI before disseminating it to neighboring devices. The perturbed information then influences routing and aggregation decisions, linking privacy protection directly to communication and energy-management behavior. Reducing the accuracy of adversarial target selection does not, however, prevent malicious traffic, and preserving routing utility under perturbation requires experimental assessment.

The scope of this study is organized around three aspects:

- The description of coordinated parent-selection and aggregation rules that account for both route-level energy availability and the distinct energy objectives of conventional and sustainable nodes.
- The examination of privacy-preserving EI dissemination as a means of reducing energy-assisted targeting accuracy, together with its implications for energy-aware network decisions.
- The presentation of a prototype evaluation under normal and adversarial conditions, incorporating nonlinear battery modeling and real indoor light-intensity traces to represent harvesting by sustainable nodes.

These aspects distinguish the system-level focus of the study from the individual techniques on which it relies. Any additional algorithmic or implementation contribution beyond the original DEPRONN-ES framework must be assessed separately from the established framework and supported by corresponding evidence.

The remainder of this article is organized as follows. Section 2 reviews the relevant literature, Section 3 formulates the energy-management problem in partially sustainable IoT networks, Section 4 presents the threat model for energy-depletion attacks, and Section 5 describes the DEPRONN-ES framework and its principal mechanisms. Section 6 presents and discusses the experimental results, and finally, Section 7 summarizes the findings, limitations, and directions for future research.

## 2. Related Work

### 2.1. Privacy-Preserving EI in Smart-City IoT Networks

Differential privacy has been investigated as a means of safeguarding energy-related data in smart-city services. For residential smart metering, Hossain et al. [22] developed a protection scheme in which a rechargeable storage unit provided the physical basis for privacy-preserving perturbations. Coordinated charging and discharging alter the externally observed demand profile, thereby obscuring household-level consumption while maintaining the usefulness of aggregated measurements. The study confirms the potential of differential privacy for protecting residential electricity data, but it does not address packet communication, route formation, or energy-resource allocation among constrained IoT nodes.

Federated Learning (FL) has also been explored in conjunction with differential privacy to protect sensitive information in distributed smart-city IoT environments. Rather than transmitting raw device data to a centralized server, FL enables participating nodes to train models locally and share only model parameters or updates for global aggregation. However, these updates may still reveal sensitive information; therefore, FL alone does not provide a complete privacy guarantee and is often combined with differential privacy. Pan et al. [23] for example, integrated FL with differential privacy to support adversarial-device detection while protecting energy-information exchanges in smart-city systems. Their approach collaboratively trained a malicious-device detector using locally observed energy-consumption behavior while perturbing the shared information with privacy noise tailored to individual devices. This is particularly relevant because battery state of charge and device-level energy consumption may expose energy-related behavior to an adversary.

Although the approaches in Hossain et al. [22] and Pan et al. [23] demonstrate the value of differential privacy for smart metering and malicious-device detection, neither addresses the network-level coexistence of conventional battery-powered and energy-harvesting nodes. They also do not investigate how privacy-preserving EI can support parent selection, traffic forwarding, and data aggregation in partially sustainable low-power networks.

### 2.2. Secure and Energy-Aware Routing in Low-Power IoT Networks

A second research stream addresses routing security and energy-aware communication in constrained IoT networks. Nandhini et al. [24] introduced Rank Attack Detection (RAD), a mechanism developed for the IPv6 Routing Protocol for Low-Power and Lossy Networks (RPL). RAD modifies RPL control messages by adding a field that supports an integer-based hash function for the detection of malicious rank manipulation, triggering an alarm. The approach seeks to improve routing security while avoiding the overhead associated with conventional cryptographic hash algorithms, but it is designed for conventional battery-powered devices and does not account for the distinct energy objectives of rechargeable energy-harvesting nodes.

Ni et al. [25] investigated a distinct setting in which an energy-harvesting receiver is powered by a rechargeable battery with non-negligible internal resistance. Their design combines a dual-path architecture with an optimization strategy that increases the volume of information successfully recovered by the receiver, measured by the number of decoded bits across different operating scenarios. Although the model accounts for harvested energy and non-ideal battery behavior, it does not represent a shared network containing both sustainable devices and conventional nodes with non-replenishable batteries. Consequently, studies Nandhini et al. [24] and Ni et al. [25] focus on different ends of the energy-source spectrum, but neither coordinates the concurrent operation of both node categories in a heterogeneous IoT deployment.

Nguyen et al. [26] introduced EHARA, an energy-harvesting-aware routing method that evaluates candidate paths through metrics reflecting transmission expenditure, incoming harvested power, and the remaining battery reserve. The algorithm accommodates multiple ambient energy sources, such as solar radiation, motion, and radio-frequency signals. Like DEPRONN-ES, EHARA favors upward routes with stronger energy availability. Nevertheless, its network model presumes harvesting-enabled devices and does not provide separate management policies for rechargeable nodes and legacy devices powered by non-replenishable batteries. The method also leaves routing-related EI unprotected, allowing an adversary to observe or misuse the reported values.

Said et al. [27] developed an Energy Management Scheme (EMS) for heterogeneous energy-constrained IoT networks. The EMS uses data aggregation and scheduling to reduce transmissions and improve energy utilization and introduces fault-tolerance strategies tailored to devices powered by conventional and renewable energy sources. The differentiation between energy-source types is relevant to partially sustainable networks, but EMS relies on centralized control and is evaluated using the NS-2 simulator, which provides limited representation of the device-level energy behavior of resource-constrained IoT hardware. The study also did not address EI privacy or energy-oriented adversaries.

Sadek et al. [28] developed Hy-IoT, a hybrid communication protocol intended for IoT deployments containing both renewable-powered and conventional devices. Its architecture separates participants into two tiers: an upper tier formed by smartphones and intelligent controllers, and a standard tier comprising sensors, actuators, and radio-frequency identification equipment. LEACH and SEP are employed to organize clustering and assign communication roles within these tiers. Although the protocol acknowledges energy-source heterogeneity and includes rechargeable devices, replenishment mainly occurs through the electrical grid rather than through ambient harvesting. Hy-IoT is therefore less suitable for sustainable sensor networks in which the available renewable energy varies dynamically with environmental conditions.

Collectively, these studies demonstrate that routing, aggregation, and scheduling can improve the energy efficiency and security of low-power IoT networks. However, they generally focus on conventional nodes, energy-harvesting nodes, or heterogeneous hardware classes separately. None of the reviewed routing approaches jointly support distinct energy-consumption profiles and privacy-preserving EI exchange under an explicit energy-depletion threat model.

### 2.3. Heterogeneous Energy Supplies and Dual-Battery Architectures

Studies of dual-battery configurations also offer relevant perspectives on coordinating dissimilar energy resources. Sharanya et al. [29] developed a battery-switching method supported by a mixed-integer linear programming model and a heuristic control policy. Their system represents sensing devices powered by two batteries and accounts for battery imperfections that can influence coverage duration and overall network longevity. The method schedules transitions between the available batteries, maintaining sensing coverage while prolonging the useful operating period of the network.

Bathre et al. [30] similarly investigated energy management using a hybrid energy-harvesting system and a dual-battery power supply. Their framework combines renewable or otherwise available power sources with a Constant Current–Constant Voltage charging mechanism. An Adaptive Neuro-Fuzzy Inference System (ANFIS) controller regulates the charging process and manages energy consumption according to battery conditions.

Dual-battery architecture represents an important form of energy heterogeneity because renewable and conventional energy can be available within the same device. This differs from a partially sustainable IoT network in which separate nodes have different battery technologies, harvesting capabilities, and communication responsibilities. While the approaches by Sharanya et al. [29] and Bathre et al. [30] primarily optimize battery behavior within individual devices, they do not address network-level routing and aggregation among heterogeneous nodes or consider malicious devices that exploit shared EI to launch energy-depletion attacks.

### 2.4. Research Gap and Positioning of DEPRONN-ES

The reviewed literature demonstrates considerable progress in energy management, routing, security, and privacy for IoT networks. The studies discussed in Section 2.1, Section 2.2 and Section 2.3 address different subsets of these requirements. Privacy-oriented studies protect energy or consumption information but do not incorporate heterogeneous energy-aware routing or data aggregation [22,23]. Routing-oriented approaches improve energy efficiency or routing security but generally do not protect the energy information (EI) used for network decisions or assume only one principal energy-source category [24,25,26,27,28]. In contrast, dual-battery approaches consider heterogeneous power supplies primarily at the individual-device level rather than coordinating conventional battery-powered and energy-harvesting nodes across a shared network [29,30].

Energy-aware routing, data aggregation, differential privacy, RPL, and energy harvesting are established techniques. The research challenge motivating their integration concerns the interaction between energy-aware decisions and the exposure of the information supporting those decisions. More accurate EI can assist routing and aggregation, but it can also help an adversary identify energy-constrained targets. Perturbing that information introduces a corresponding trade-off between privacy protection and decision accuracy.

Table 1 summarizes these approaches according to six capabilities that are particularly important for partially sustainable smart-city IoT networks: heterogeneous energy-source support, energy-aware routing, data aggregation, EI privacy, explicit adversarial modeling, and evaluation in resource-constrained IoT environments. The comparison emphasizes the extent to which each study addresses these requirements rather than reproducing methodological details already discussed in the preceding subsections.

As shown in Table 1, no reviewed approach simultaneously addresses all six requirements. Hossain et al. [22] and Pan et al. [23] demonstrate the usefulness of differential privacy for protecting energy- or consumption-related information, but neither incorporates privacy-preserving EI into routing and aggregation decisions for partially sustainable IoT networks. Nandhini et al. [24] considers adversarially resilient routing in a constrained-device environment but focuses on conventional battery-powered nodes and does not protect shared EI. Nguyen et al. [26] introduces energy-harvesting-aware routing but assumes harvesting-enabled devices and leaves routing-related energy information unprotected. Said et al. [27] and Sadek et al. [28] acknowledge heterogeneous energy sources, but neither combines this capability with EI privacy and explicit protection against energy-oriented attacks. Finally, Sharanya et al. [29] and Bathre et al. [30] manage heterogeneous energy supplies within individual devices rather than coordinating distinct energy profiles across networked IoT nodes.

The original DEPRONN-ES framework brings these concerns together by coordinating dual energy profiles, parent selection, aggregation, and privacy-preserving EI dissemination. Its Dual Energy Profile Mechanism differentiates the energy-management objectives of conventional and harvesting-enabled nodes. Route-level energy information supports forwarding and aggregation decisions, while privacy perturbation is intended to reduce the accuracy of energy-assisted adversarial targeting. These mechanisms interact because perturbation can also change the energy estimates used for legitimate network decisions.

The present study examines this coordinated operation rather than claiming a new individual routing, aggregation, or differential-privacy technique. The integration itself is attributed to the original DEPRONN-ES framework. The questions considered here concern the extent to which perturbed EI reduces targeting accuracy and the implications for energy management and routing utility. Establishing the benefit attributable to each component requires matched baseline and ablation comparisons; the functional coverage summarized in Table 1 cannot substitute for those experiments.

## 3. Energy-Management Problem Formulation

**Figure 2:**
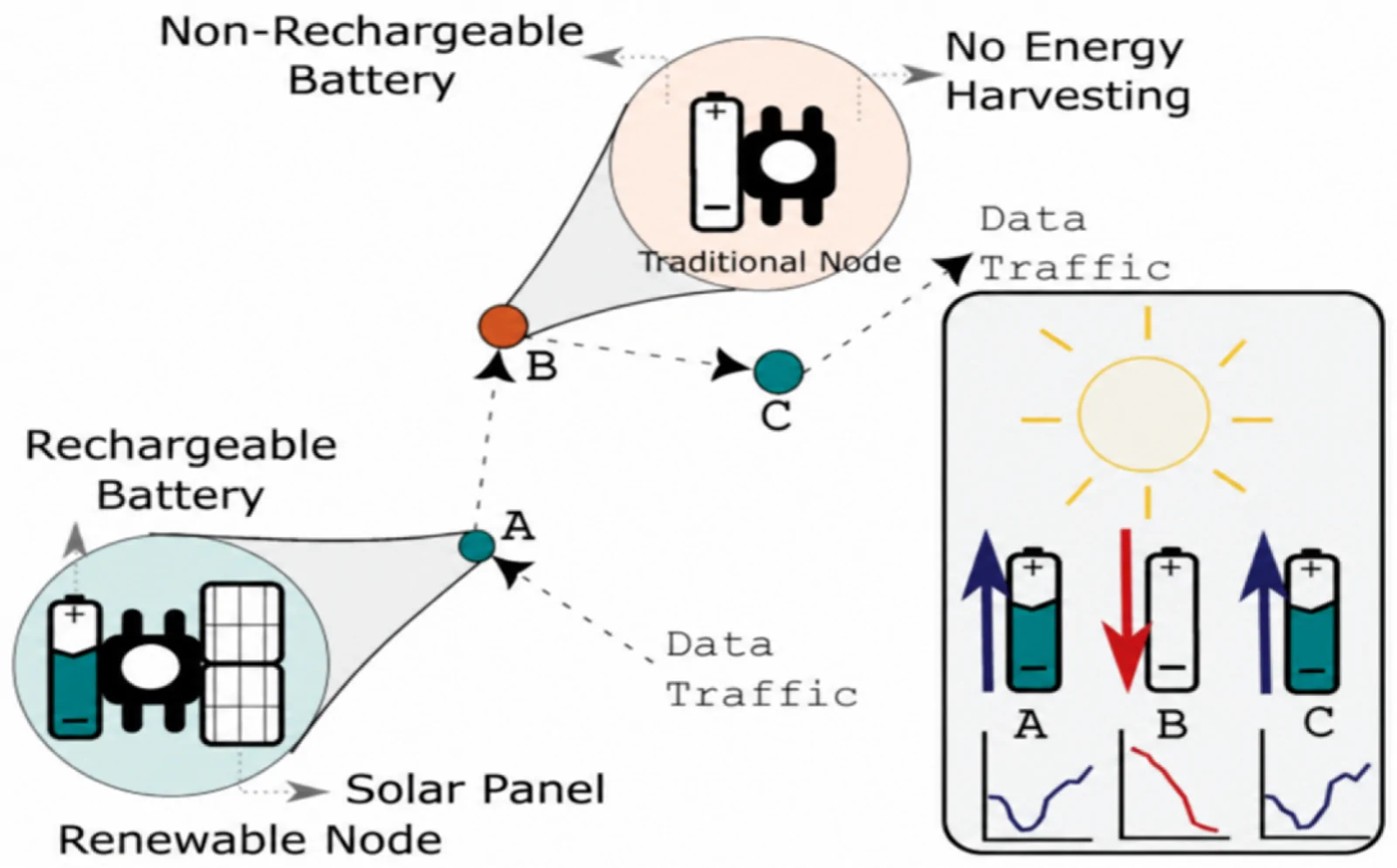
Conflicting energy-consumption profiles in a partially sustainable IoT network.

Figure 2 depicts a three-device IoT topology containing two energy-source categories. Devices A and C obtain ambient energy through harvesting units and store it in replenishable batteries, whereas device B relies exclusively on a finite, non-rechargeable power cell. All three devices participate in exchanging and forwarding network traffic, thereby representing a partially sustainable deployment.

The two device categories follow different and potentially competing power-management objectives. Energy-harvesting nodes can expend the renewable energy they collect, provided that their long-term consumption remains consistent with energy-neutral operation. Favorable harvesting conditions increase their usable energy reserve, allowing them to undertake a greater share of sensing, packet relaying, and data aggregation. Conventional nodes, in contrast, must conserve charge throughout their operation because depleted batteries cannot recover autonomously. Reconciling these distinct priorities requires the network’s energy management to balance multiple goals at once.

The network topology is modeled as a graph $G=\left(N,E\right)$, where N and E denote the sets of nodes and wireless links, respectively. The node set is defined as N=B∪S, where B is the set of gateway nodes and S is the set of resource-constrained IoT nodes. The network contains a single gateway b∈B, which is assumed to have a continuous external energy supply.

All nodes have the same nominal wireless communication range R. A wireless link exists between nodes i,j∈N if and only if their Euclidean distance satisfies $d_{ij}\leq R$. The resource-constrained nodes are divided into two disjoint subsets, $S=N_{r}\cup N_{n}$ and $N_{r}\cap N_{n}=⌀$, where $N_{r}$ represents sustainable nodes powered by rechargeable batteries and energy-harvesting components, while $N_{n}$ represents traditional nodes powered by non-rechargeable batteries. The nodes may have heterogeneous initial battery levels. Their geographical positions are assumed to be static, as commonly observed in smart-metering, smart-grid, environmental-monitoring, and smart-parking monitoring.

A bounded operating interval is discretized into the set t∈{0,1,…,T}, where T<∞, t=0 denotes the initial system state, and each time slot has a fixed duration Δ.

A node can operate in one of four states during each time slot: sleeping z, sensing s, transmitting Tx, or receiving Rx. Accordingly, the set of possible operating states is M={z,s,Tx,Rx}.

Let $S_{i}^{m}\left(t\right)$ be a binary variable that indicates whether node i operates in state m∈M during time slot t. Specifically, $S_{i}^{m}\left(t\right)=1$ if node i is in state m, and $S_{i}^{m}\left(t\right)=0$ otherwise. A node can occupy only one operating state in each time slot. Therefore,

(1) $$\begin{array}{cc} \sum_{m\in M}S_{i}^{m}\left(t\right) & =S_{i}^{z}\left(t\right)+S_{i}^{s}\left(t\right)+S_{i}^{Tx}\left(t\right)+S_{i}^{Rx}\left(t\right)=1,\\ \left(i,t\right) & \in S\times \{1,\ldots ,T\}. \end{array}$$

The energy required by node i∈S to perform a sensing operation is represented by the constant $C_{s}$. This cost is assumed to be identical across the network because the nodes employ similar sensing hardware.

Communication energy depends on both the direction of communication and the amount of exchanged data. Let $C_{Tx}^{p}$ and $C_{Tx}^{h}$ denote the energy required to transmit one payload and one header, respectively. Similarly, $C_{Rx}^{p}$ and $C_{Rx}^{h}$ denote the energy required to receive one payload and one header. Let $p_{ij}\left(t\right)$ and $h_{ij}\left(t\right)$ represent the numbers of payloads and headers transmitted over link $\left(i,j\right)\in E$ during time slot t. The corresponding link-level communication costs are:

(2) $$\begin{array}{rr} c_{ij}^{Tx}\left(t\right) & =C_{Tx}^{p}p_{ij}\left(t\right)+C_{Tx}^{h}h_{ij}\left(t\right),\\ c_{ij}^{Rx}\left(t\right) & =C_{Rx}^{p}p_{ij}\left(t\right)+C_{Rx}^{h}h_{ij}\left(t\right). \end{array}$$

All resource-constrained nodes have finite-capacity batteries. The residual battery energy of node i at time slot t is represented by $B_{i}\left(t\right)$, while $B_{i}^{max}$ denotes its maximum battery capacity. Therefore, $0\leq B_{i}\left(t\right)\leq B_{i}^{max}$ for every i∈S and 0≤t≤T.

Let $C_{i}\left(t\right)$ denote the total energy consumed by node i during time slot t. Each operating state has a distinct energy-consumption profile. In the sleeping state, the node consumes a small and constant amount of energy, $S_{i}^{z}\left(t\right)C_{z}$, where $C_{z}$ denotes the sleeping-state energy cost. In the sensing state, the corresponding cost is $S_{i}^{s}\left(t\right)C_{s}$, where $C_{s}\gg C_{z}$ generally holds.

In the transmission and reception states, energy consumption consists of two components: a fixed radio-activation cost and a variable cost determined by the numbers of exchanged headers and payloads. Let $C_{Tx}$ and $C_{Rx}$ denote the fixed costs of activating the radio in transmission and reception modes, respectively. For compactness, $C_{i}^{Tx}\left(t\right)$ denotes the complete transmission-state cost. The energy consumed by node i while transmitting is calculated as:

(3) $$\begin{array}{cc} C_{i}^{Tx}\left(t\right) & =C_{Tx}+C_{Tx}^{p}p_{i}^{out}\left(t\right)+C_{Tx}^{h}h_{i}^{out}\left(t\right),\\ C_{i}\left(t\right) & =S_{i}^{Tx}\left(t\right)C_{i}^{Tx}\left(t\right). \end{array}$$

Similarly, $C_{i}^{Rx}\left(t\right)$ denotes the complete reception-state cost, and the energy consumed while receiving is:

(4) $$\begin{array}{cc} C_{i}^{Rx}\left(t\right) & =C_{Rx}+C_{Rx}^{p}p_{i}^{in}\left(t\right)+C_{Rx}^{h}h_{i}^{in}\left(t\right),\\ C_{i}\left(t\right) & =S_{i}^{Rx}\left(t\right)C_{i}^{Rx}\left(t\right). \end{array}$$

The total numbers of outgoing and incoming headers associated with node i are defined as:

(5) $$h_{i}^{out}\left(t\right)=\sum_{j:\left(i,j\right)\in E}h_{ij}\left(t\right),\;\;h_{i}^{in}\left(t\right)=\sum_{j:\left(j,i\right)\in E}h_{ji}\left(t\right).$$

Likewise, the total numbers of outgoing and incoming payloads are:

(6) $$p_{i}^{out}\left(t\right)=\sum_{j:\left(i,j\right)\in E}p_{ij}\left(t\right),\;\;p_{i}^{in}\left(t\right)=\sum_{j:\left(j,i\right)\in E}p_{ji}\left(t\right).$$

### 3.1. Energy Optimization for Traditional Nodes

Traditional nodes $i\in N_{n}$ depend entirely on non-rechargeable batteries. The energy consumed by these nodes cannot be recovered; therefore, their primary objective is to minimize cumulative energy consumption over the entire operational horizon. This objective is formulated as:

(7) $$minF_{1}=\sum_{i\in N_{n}}\sum_{t=1}^{T}C_{i}\left(t\right).$$

Minimizing $F_{1}$ extends the operational lifetime of traditional nodes and reduces the probability of network partitioning caused by battery depletion. Under the discrete-time formulation, the scheduler assigns operating modes and communication tasks to each device across successive slots with the aim of reducing the energy expenditure of conventional nodes. Accordingly, when suitable sustainable devices are available, traditional nodes with depleted battery reserves should undertake a smaller share of packet forwarding and data aggregation.

### 3.2. Energy Management of Energy-Harvesting Nodes

For sustainable nodes $i\in N_{r}$, the energy harvested from ambient sources must be incorporated into the energy-management model. Let $H_{i}\left(t\right)\geq 0$ denote the energy harvested by node i during time slot t.

This study assumes that $H_{i}\left(t\right)$ is deterministic, which is appropriate when measured energy traces or short-term harvesting predictions are available. Representing harvested energy as a stochastic variable would require a stochastic optimization formulation and would substantially increase the computational complexity of the problem.

The battery level of a rechargeable node must remain within its operational limits:

(8) $$B_{i}^{min}\leq B_{i}\left(t\right)\leq B_{i}^{max},\;\;\forall i\in N_{r},\;1\leq t\leq T.$$

The evolution of its battery level is expressed as:

(9) $$B_{i}\left(t\right)=B_{i}\left(t-1\right)-C_{i}\left(t\right)+H_{i}\left(t\right),\forall i\in N_{r},\;1\leq t\leq T.$$

Equation (9) represents the charging and discharging behavior of a rechargeable node: its battery level decreases according to the consumed energy and increases according to the harvested energy. This linear energy-balance model serves as a theoretical simplification for the problem formulation and does not explicitly represent nonlinear battery dynamics. In the Renode-based experimental evaluation, battery behavior is modeled using the nonlinear Kinetic Battery Model (KiBaM), which represents available- and bound-charge dynamics and accounts for battery recovery effects.

To ensure energy-neutral operation over the considered horizon, the final battery level of each sustainable node must not be lower than its initial level:

(10) $$B_{i}\left(T\right)\geq B_{i}\left(0\right),\forall i\in N_{r}.$$

Constraint (10) ensures that a sustainable node does not experience a net battery-energy loss over the complete operational horizon. Together, constraints (8)–(10) prevent battery depletion, avoid exceeding battery capacity, and support sustainable long-term operation.

For nodes equipped with rechargeable batteries, the objective of optimization is to reduce the accumulated gap between harvested energy and energy expenditure:

(11) $$minF_{2}=\sum_{i\in N_{r}}\sum_{t=1}^{T}\left[H_{i}\left(t\right)-C_{i}\left(t\right)\right].$$

Under the energy-neutrality constraint in (10), the cumulative difference in (11) is non-negative. Minimizing it encourages sustainable nodes to use available harvested energy instead of leaving it unused, particularly when their batteries approach maximum capacity. Consequently, a scheduler can assign additional sensing, forwarding, or data-aggregation responsibilities to sustainable nodes when sufficient harvested energy is available.

### 3.3. Dual Energy-Consumption Problem

The proposed energy-management formulation involves multiple objectives, as it accounts for the energy-conservation requirement $F_{1}$ for traditional nodes and the energy-balancing objective $F_{2}$ for sustainable nodes. The problem is formulated using lexicographic optimization:

(12) $$\underset{x\in \Omega }{lex\;min}\left(F_{1}\left(x\right),F_{2}\left(x\right)\right).$$

Here, x represents the network-management decisions, including node-state scheduling, parent-node selection, traffic forwarding, and data-aggregation levels. The set Ω represents the feasible decision space defined by constraints (1)–(10).

Lexicographic optimization arranges the objective functions according to their relative importance and solves them sequentially. In (12), the energy-conservation objective for traditional nodes, $F_{1}$, is assigned the highest priority. This ordering is necessary because a traditional node cannot return to normal operation after its non-rechargeable battery is depleted. The second objective, $F_{2}$, concerns sustainable nodes, whose depleted reserves can subsequently be replenished using energy collected from ambient sources.

A scheduler-based solution could determine the operating states and communication responsibilities of each node to address the formulation in (12). However, the computational complexity of finding the optimal solution increases significantly with the numbers of nodes, links, time slots, and routing alternatives. A centralized solution would also require the periodic collection of battery states, harvesting measurements, and traffic information from the entire network. This process would introduce considerable communication and computational overhead and could reveal sensitive EI. To avoid this difficulty, this study adopts a distributed heuristic that can be embedded directly within a hierarchical routing process. The resulting mechanism allows conventional and energy-harvesting devices to follow their respective power-management objectives while cooperating in a shared network topology. Since the energy data needed for these decisions could expose resource-constrained devices to targeted attacks, the framework also protects the energy values exchanged between neighboring nodes.

## 4. Threat Model

A malicious node located within the communication range of legitimate IoT devices can exploit the EI exchanged in a smart-city network. By collecting information such as battery type and state of charge, the attacker can identify energy-constrained nodes and selectively target them with energy-depletion traffic. Disabling these nodes may interrupt network communication and compromise the availability of the smart-city services that depend on them.

### 4.1. Adversary Capabilities and Assumptions

The threat model assumes a malicious-but-covert adversary that seeks to exhaust the battery reserves of either conventional or energy-harvesting IoT devices without revealing its presence. Rather than injecting malformed or readily detectable packets, the attacker produces an excessive stream of protocol-compliant messages that resemble normal network traffic. Consequently, the victim consumes additional energy while receiving, processing, and relaying these unnecessary messages.

The adversary may be either internal or external. An internal adversary is a compromised or malicious IoT node with legitimate access to the information exchanged within the network, and it can directly receive the control messages and energy announcements transmitted by neighboring nodes. An external adversary is a device that has obtained access to the wireless network or can communicate with legitimate nodes after circumventing the applicable access-control mechanisms. In both cases, the attacker is assumed to be within communication range of the targeted nodes and capable of receiving their periodically announced EI.

The attacker does not necessarily compromise the cryptographic mechanisms or physically manipulate the targeted devices but instead exploits the legitimate network functions available to an authorized or apparently legitimate node, making the attack difficult to distinguish from authorized normal activity.

The scope of the threat model is limited to EI-assisted energy-depletion attacks. Other attacks, including Sybil, rank-manipulation, sinkhole, wormhole, and routing-table attacks, are outside the scope of this study. The adversary is also not assumed to alter the physical energy-harvesting process. Its objective is specifically to use disclosed EI to identify vulnerable nodes and accelerate their energy depletion.

### 4.2. Energy-Depletion Attack Process

Energy-aware routing mechanisms commonly require nodes to periodically announce their residual battery levels to neighboring devices. This information supports route selection and workload distribution, but it can also expose nodes with limited energy reserves.

The attack considered consists of three main stages. First, the malicious node monitors the EI announcements exchanged by its neighbors. These announcements may disclose the residual battery level, battery type, or energy-harvesting status of each node. Second, the adversary analyzes the collected information and selects the node considered most vulnerable. A traditional node with a low residual battery level represents an attractive target because its energy cannot be replenished. A sustainable node may also become vulnerable when its battery level and current harvesting rate are insufficient to support an increased workload. Third, after selecting its target, the attacker generates unnecessary but legitimate-looking traffic. The victim must receive, process, respond to, aggregate, or forward these messages, each time activating the node’s radio, processor, or sensing components and thereby increasing its energy consumption. Because the packets conform to the network protocol, the malicious activity may remain unnoticed until the victim’s battery level has been substantially reduced.

Unlike indiscriminate flooding, the considered adversary uses EI to concentrate its traffic on the node most likely to fail, thereby achieving a greater effect with fewer malicious messages and reducing the probability of detection. Periodic EI announcements also allow the attacker to update its target selection as the energy conditions of the network change.

### 4.3. Impact on Smart-City Services

Successful energy-depletion attacks can considerably reduce network lifetime and reliability. When a traditional node exhausts its non-rechargeable battery, it can no longer sense, receive, or forward data. If that node occupies an important position in the routing tree, its failure may disconnect multiple descendant nodes and require the network to reconstruct its routing topology. This process creates additional control of traffic and energy consumption.

Although sustainable nodes can replenish their batteries, they are not immune to energy-depletion attacks. An attack may temporarily increase their consumption beyond the available harvested energy, causing their batteries to reach the minimum operational level. Consequently, the affected nodes may suspend sensing and communication activities until sufficient energy has been harvested.

In smart-city environments, such failures may disrupt services including traffic monitoring, smart parking, smart metering, environmental monitoring, and predictive infrastructure maintenance [31,32,33]. The consequences can include missing sensor readings, delayed control decisions, reduced service availability, network partitioning, and increased device-maintenance costs.

The threat addressed by DEPRONN-ES is therefore not the transmission of EI itself, which is required for energy-aware routing, but the disclosure of sufficiently accurate EI for reliable target selection. The proposed differential-privacy mechanism perturbs the announced EI to reduce the adversary’s ability to identify the most vulnerable node. DEPRONN-ES does not directly prevent the generation of malicious traffic but rather reduces the effectiveness of EI-assisted target selection and, consequently, the expected impact of energy-depletion attacks.

## 5. DEPRONN-ES: Dual Energy Profiles and Privacy-Preserving EI

### 5.1. Proposed DEPRONN-ES Framework

DEPRONN-ES is designed to accomplish two complementary goals: accommodate the contrasting energy-management requirements of sustainable and conventional devices within a unified IoT deployment and reduce the security risk associated with revealing sensitive EI. The framework addresses these goals through the DEPM, which coordinates node behavior according to the available power source, and a differential-privacy mechanism that protects the numerical energy values exchanged across the network.

#### 5.1.1. DEPM

**Figure 3:**
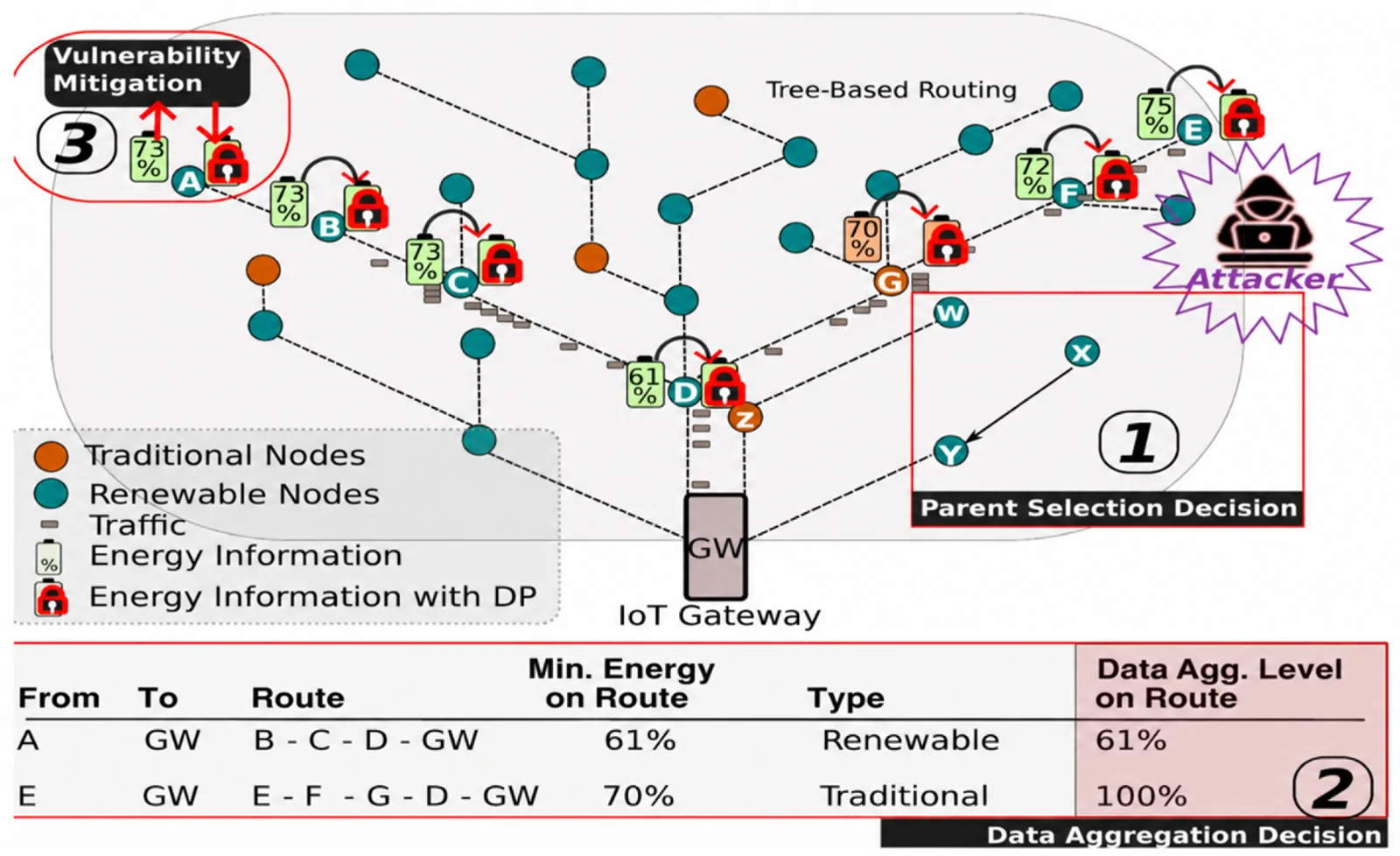
Overview of the DEPRONN-ES parent-selection, data-aggregation, and privacy-preserving mechanisms.

As illustrated in Figure 3, DEPRONN-ES is designed for tree-based routing networks in which IoT devices forward their traffic toward a gateway. Each traditional or sustainable node makes two energy-aware decisions. First, it selects a neighboring node as its preferred parent for forwarding traffic toward the IoT gateway. Second, it determines the data-aggregation level applied to the traffic passing through it.

Parent-selection rule: For each node, DEPRONN-ES selects the candidate parent that maximizes the advertised estimate of the minimum energy available along the path to the gateway, while prioritizing fully sustainable routes over routes containing traditional nodes.

Let $P_{i}$ denote the set of candidate parents available to node i. Each candidate announces a PathEnergy value representing the minimum available energy along its path to the gateway. Consequently, the preferred parent is selected according to the maximum bottleneck energy:

(13) $$p_{i}^{*}=arg\underset{p\in P_{i}}{max}PathEnergy\left(p\right)$$

subject to the preference for a fully sustainable path whenever one is available. This max-min decision avoids selecting a route containing a critically energy-constrained node.

In the example shown in Figure 3, node X has two candidate parents, Y and W. Selecting Y produces a path composed entirely of sustainable nodes. By contrast, selecting W produces a route that contains traditional node Z. DEPRONN-ES therefore selects Y because fully sustainable paths receive priority over paths that rely on non-rechargeable batteries. If multiple fully sustainable paths are available, the path with the highest minimum energy value is selected.

Traditional nodes contribute their residual energy to the bottleneck calculation rather than a zero-valued profile indicator. Route composition is represented separately by the routeContainsTraditional flag. A traditional node sets this flag to true, while a sustainable node inherits the flag announced by its selected parent. Consequently, two traditional candidate parents can advertise different PathEnergy values when their route bottlenecks differ. Their values may nevertheless be similar or equal if they share a downstream bottleneck, and differential-privacy noise may alter their ordering. The metric therefore compares estimated route bottlenecks rather than only the candidate parents’ local battery levels.

Data-aggregation rule: DEPRONN-ES determines the aggregation level associated with a route according to its minimum available energy and the energy-source types of the nodes located along that route.

Data aggregation reduces the number of transmitted messages by combining multiple payloads into a single packet, but it also requires buffering and processing at intermediate nodes. DEPRONN-ES therefore adapts the aggregation level to the energy profile of the selected route.

Consider a path from node A to the gateway through nodes B, C, and D. If all these nodes are sustainable and node D has the lowest energy level, the aggregation level is determined using the energy announced by D. For example, a normalized minimum path-energy value of 40% results in a 40% aggregation level under the mapping implemented in Algorithm 1.
**Algorithm 1.** DEPRONN-ES executed by node iInput: Candidate parent set P_i; local energy E_i; battery type Type_i; privacy budget ε; sensitivity ΔfOutput: Preferred parent p_i*; private path-energy value; data-aggregation level1:Initialize candidateSet ← empty2:Start3:while PathEnergy announcements are received do4:Store candidate parent, PathEnergy, and routeContainsTraditional flag5:end while6:if a fully sustainable candidate path exists (candidate.routeContainsTraditional = false) then7:p_i* ← candidate with maximum PathEnergy among those whose routeContainsTraditional flag is false8:else9:  p_i* ← candidate with maximum PathEnergy10:end if11:maxPathEnergy ← PathEnergy announced by p_i*12:if Type_i = Traditional then13:localContribution ← E_i; routeContainsTraditional ← true14:else15:localContribution ← E_i; routeContainsTraditional ← flag announced by p_i*16:end if17:pathEnergy ← min(localContribution, maxPathEnergy)18:privatePathEnergy ← DiffPrivacy(pathEnergy, Δf, ε)19:Broadcast privatePathEnergy and routeContainsTraditional to neighboring nodes20:dataAggLevel ← AdjustDataAggregation(pathEnergy, routeContainsTraditional)21:End22:procedure DiffPrivacy(energy, Δf, ε)23:  Draw U uniformly from (−0.5, 0.5)24:  noise ← −(Δf/ε) × sign(U) × ln(1 − 2|U|)25:  privateEnergy ← energy + noise26:  return MapToValidEnergyRange(privateEnergy)27:end procedure29:procedure AdjustDataAggregation(pathEnergy, routeContainsTraditional)30:  normalizedEnergy ← Normalize(pathEnergy)31:if routeContainsTraditional = true then32:    dataAggLevel ← 100%33:  else34:    dataAggLevel ← EnergyToAggregationFunction(normalizedEnergy)35:  end if36:  return dataAggLevel37:end procedure

Consider another route from node E to the gateway through nodes F, G, and D. Assume that node D is sustainable and has 61% remaining energy, whereas node G is traditional and has 70% residual battery capacity. Although node G currently has more energy, its battery cannot be replenished. DEPRONN-ES therefore assigns the maximum aggregation level of 100% to reduce the number of packets that node G must transmit and receive.

#### 5.1.2. Privacy-Preserving EI Announcements

Raw residual-energy measurements create privacy and security risk because they can reveal which nodes are most susceptible to energy-depletion attacks. DEPRONN-ES addresses this vulnerability by applying differential privacy before numerical EI is announced. Controlled random noise is added to the PathEnergy value, reducing the adversary’s ability to infer the exact battery condition of an individual node.

The mechanism uses the Laplace distribution, whose scale is determined by the sensitivity Δf and privacy budget ε. Sensitivity represents the maximum possible change in the released query under the adopted adjacency definition:

(14) $$\Delta f=\underset{D∼D^{\prime }}{max}\left|f\left(D\right)-f\left(D^{\prime }\right)\right|$$

where $f\left(\cdot \right)$ represents the released energy query and $D∼D^{\prime }$ denotes two adjacent energy inputs.

The privacy budget ε controls the privacy–utility trade-off. A smaller value of ε generates more noise and provides stronger privacy, but it may reduce the accuracy of routing and aggregation decisions. A larger value introduces less noise and preserves greater utility, but it provides weaker privacy protection. Selecting an appropriate value therefore requires target-selection resistance to be balanced with the accuracy required for energy-aware routing.

For each announcement, DEPRONN-ES generates a random variable:

(15) $$U∼Uniform\left(-\frac{1}{2},\frac{1}{2}\right)$$

and calculates Laplace noise as

(16) $$\eta =-\frac{\Delta f}{\varepsilon }sgn\left(U\right)ln\left(1-2\left|U\right|\right).$$

The private energy announcement is then obtained from

(17) $$\tilde{E}=E+\eta$$

where E is the path-energy value before perturbation and $\tilde{E}$ is the value released to neighboring nodes. The perturbed value is mapped to the valid range supported by the energy field before transmission. This mapping is a post-processing operation, so it does not weaken the differential-privacy guarantee.

As illustrated in Figure 3, nodes A–G announce perturbed EI. Perturbing EI is intended to reduce the accuracy of adversarial target selection; however, observations accumulated across multiple announcements may improve an attacker’s estimates. The routing protocol can still use the approximate values for distributed energy-aware decisions, but the accuracy of adversarial target selection is reduced.

Repeated EI announcements create a limitation under persistent observation. An adversary collecting multiple DIO messages from the same node may reduce the effect of independently sampled Laplace noise through temporal averaging, particularly when the underlying path-energy value changes slowly. Although harvesting and consumption cause energy values to vary, these dynamics do not eliminate the risk of temporal inference. A per-announcement privacy budget therefore must not be interpreted as an equivalent privacy guarantee for the complete 72-h sequence.

Potential mitigations include allocating a total privacy budget across releases, limiting the number of fresh energy disclosures, and using mechanisms designed for differential privacy under continual observation. Repeated DIO messages could also reuse a previously released perturbed value between scheduled energy updates, reducing fresh disclosures at the cost of less current routing information. These measures require explicit privacy accounting and evaluation of their impact on routing performance. Resistance to persistent temporal-averaging attacks has not been established by the reported evaluation and remains a direction for future work.

Differential privacy does not encrypt the exchanged messages or prevent an adversary from generating malicious traffic. Instead, it reduces the precision of the EI available to the attacker. Its computational cost is also lower than that of many cryptographic protection mechanisms, which can introduce substantial processing, memory, and communication overhead on constrained IoT devices.

#### 5.1.3. DEPRONN-ES Pseudocode

Algorithm 1 summarizes the distributed operation of DEPRONN-ES. Each node collects PathEnergy announcements from its candidate parents, selects its preferred parent, generates a privacy-preserving energy announcement, and determines its data-aggregation level.

Lines 1–5 collect the PathEnergy values and routeContainsTraditional flags announced by the candidate parents. Lines 6–10 implement the parent-selection rule. Fully sustainable paths receive priority, after which the candidate with the highest advertised PathEnergy within the eligible set is selected. If no fully sustainable candidate path exists, all eligible candidate parents are compared using their advertised PathEnergy values. These values are privacy-perturbed estimates, so their ordering may differ from that of the true route bottlenecks.

Lines 12–17 calculate the energy value associated with the new path. Both traditional and sustainable nodes contribute their local residual energy. A traditional node sets routeContainsTraditional to true, whereas a sustainable node inherits the flag announced by its selected parent. The new path-energy estimate is calculated as the minimum of the local energy and the selected parent’s advertised PathEnergy. This separates route composition from numerical energy availability and removes the forced-zero contribution of traditional nodes. Two traditional parents can therefore be distinguished when their advertised route-energy estimates differ, although a shared downstream bottleneck can yield equal underlying estimates.

Lines 18–19 apply the differential-privacy mechanism and broadcast the perturbed PathEnergy together with the unperturbed routeContainsTraditional flag. The flag is treated as public route-composition information. A single independent Laplace sample is generated for each announcement. Finally, line 20 and lines 29–37 determine the data-aggregation level according to the new path-energy estimate and the propagated route-type flag. Routes containing a traditional node receive an aggregation level of 100%; otherwise, the normalized path-energy estimate is mapped through EnergyToAggregationFunction.

The gateway initializes its advertised PathEnergy to the maximum valid energy value and its routeContainsTraditional flag to false. Local and advertised energy values are represented on the same normalized scale.

### 5.2. Implementation

The DEPRONN-ES prototype was implemented using Contiki-NG [34,35,36], an operating system developed for IoT devices with constrained processing, memory, and energy resources. Its functions were distributed across the routing, network, and application layers. MQTT for Sensor Networks (MQTT-SN) [37,38] supported lightweight publish–subscribe communication at the application layer, whereas RPL Lite [39,40] established IPv6 routes across low-power and lossy links. Both protocols were selected because their low-overhead operation is suitable for devices with restricted computing capacity, memory, bandwidth, and energy reserves.

#### 5.2.1. RPL-Lite and DIO Integration

RPL is a proactive routing protocol that constructs a Destination-Oriented Directed Acyclic Graph (DODAG) rooted at a gateway or another designated destination. Nodes select their preferred parents from neighboring candidates and use the resulting topology to forward traffic upward toward the DODAG root.

DODAG Information Object (DIO) messages advertise information about the RPL-lite instance, DODAG configuration, and routing rank, enabling neighboring nodes to evaluate candidate parents. These messages propagate outward from the root toward the network nodes, while the information they contain is used to construct upward routes toward the root. DEPRONN-ES extends the information carried in DIO messages so that nodes can announce their privacy-preserving PathEnergy values. The revised algorithm additionally requires each candidate to announce a routeContainsTraditional flag, which identifies whether its path to the gateway contains a non-rechargeable node.

The transmission frequency of DIO messages is controlled by the RPL-lite Trickle algorithm. Under stable conditions, Trickle gradually reduces the frequency of control-message transmissions. When an inconsistency or topology change is detected, the transmission rate increases so that updated routing information can be distributed more rapidly [41,42,43]. Because DEPRONN-ES communicates EI through DIO messages, it inherits this adaptive behavior and can respond to changes in topology and energy conditions without introducing an independent control-message mechanism [44,45].

**Figure 4:**
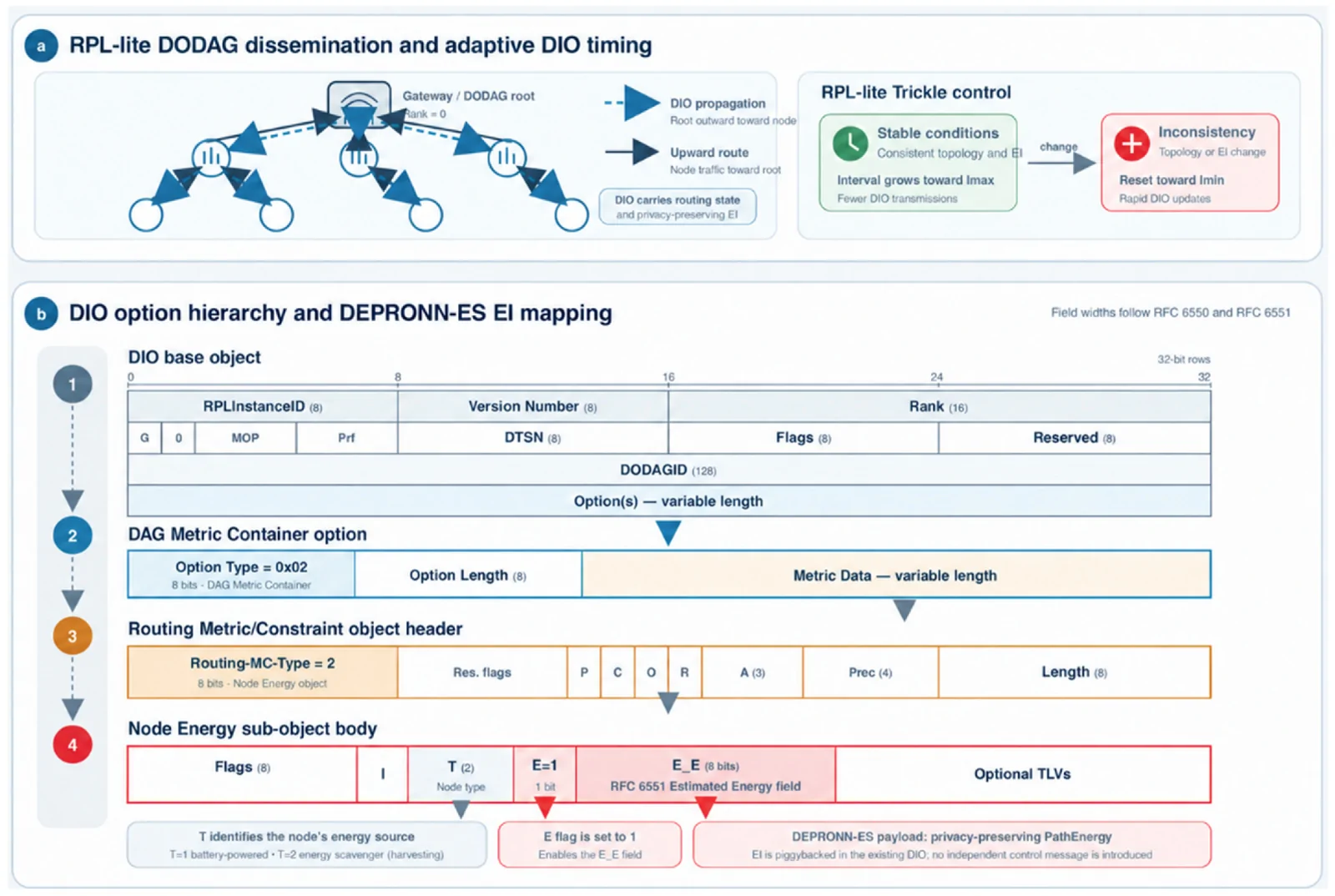
DIO message structure and the fields used to carry DEPRONN-ES EI.

The DIO message structure used in the implementation is illustrated in Figure 4, with particular emphasis on the fields responsible for routing-metric specification and node-level energy reporting.

DEPRONN-ES assigns a value of 2 to the Routing-MC-Type field in the DAG Metric Container, thereby designating a Node Energy Object as defined in RFC 6551. It also activates the E flag in the Node Energy Object by setting it to 1, which makes the 8-bit Estimated Energy (E_E) field available for reporting EI.

The E_E field was originally used to represent estimated remaining energy. DEPRONN-ES reuses this field to communicate the privacy-preserving PathEnergy value described in Algorithm 1. This is a DEPRONN-ES-specific interpretation of the numerical field as a path-energy estimate. The perturbed value is mapped and quantized to the valid encoding range before transmission.

Route composition is maintained independently of this numerical value. A traditional node sets routeContainsTraditional to true, whereas a sustainable node inherits the flag announced by its selected parent. The gateway initializes the flag to false. Consequently, a sustainable node whose selected path includes a traditional device also advertises a true flag. The flag is treated as public routing information and is not perturbed by the differential-privacy mechanism.

The revised DIO integration must carry this route-wide flag separately from the numerical E_E value. The flag must not be inferred from a zero-energy value or solely from the announcing node’s power-source type. Receiving nodes use the flag to identify fully sustainable candidate paths and then compare advertised PathEnergy values within the eligible candidate set, as specified in Algorithm 1. Carrying both items within DIO announcements avoids a separate message type; any additional encoding overhead must be included in the implementation description and evaluation.

#### 5.2.2. Battery-State Estimation

To calculate the energy values encoded in PathEnergy, the implementation integrates the Kinetic Battery Model (KiBaM) [46,47,48], which represents battery behavior using available- and bound-charge wells. It provides a more realistic representation of battery discharge and recovery than a purely linear model. Each node uses KiBaM to estimate its remaining battery energy. Sustainable nodes additionally incorporate harvested energy into their battery state, and the estimated value is then combined with the PathEnergy announced by the preferred parent to determine the minimum energy available along the new route. Differential privacy is applied before the resulting value is encoded in the E_E field and announced to neighboring nodes.

#### 5.2.3. MQTT-SN Data Aggregation

Data aggregation was enabled through a cross-layer extension to the IPv6 processing path above 6LoWPAN. During packet inspection, the implementation examines the UDP payload and the MQTT-SN message-type field to identify eligible PUBLISH messages. When a qualifying message has not yet reached the gateway, its application data are transferred to the aggregation service instead of being forwarded immediately. MQTT-SN control packets and publications that do not satisfy the aggregation criteria continue through the regular forwarding path.

The application-layer aggregation service consists of a buffer for retaining MQTT-SN publication data, a timer that determines when a combined message should be generated, and procedures for encoding and decoding aggregated records. DEPRONN-ES adjusts the timer interval and buffer threshold according to the selected aggregation level, and when either threshold is reached, the stored records are placed in the payload of a new MQTT-SN PUBLISH message and transmitted toward the gateway. To avoid interfering with acknowledgment and retransmission procedures, aggregation is restricted to QoS 0 publications directed to the same aggregation topic and gateway.

**Figure 5:**
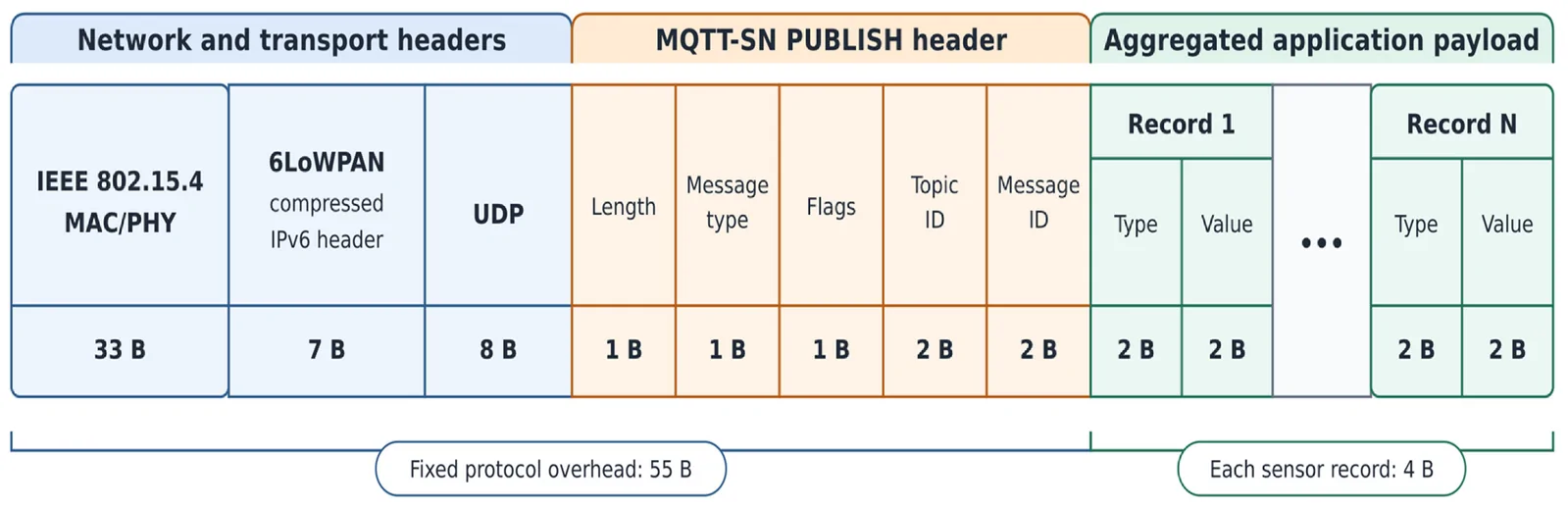
Header allocation and payload structure of an aggregated MQTT-SN PUBLISH message.

The resulting communication stack comprises IEEE 802.15.4 at the link layer, 6LoWPAN for IPv6 adaptation, RPL Lite for route establishment, UDP for transport, and MQTT-SN for publish–subscribe messaging. Figure 5 presents the header allocation across these layers and the application-specific format used to encode multiple sensor records within one MQTT-SN publication.

Each aggregated record occupies 4 bytes: 2 bytes identify the data type, and the remaining 2 bytes represent its value. The maximum number of complete records that can be included without causing 6LoWPAN fragmentation depends on the payload capacity remaining after the IEEE 802.15.4, 6LoWPAN, UDP, and MQTT-SN header overheads are deducted from the maximum IEEE 802.15.4 frame size. The remaining capacity is then divided by the 4-byte record size, and only complete records are included. Combining several measurements within a single MQTT-SN publication decreases the required number of radio transmissions and receptions, which limits communication-related energy expenditure, particularly along routes containing devices powered by non-rechargeable batteries.

## 6. Performance Evaluation

This section evaluates whether DEPRONN-ES fulfills its two principal objectives: protecting EI against targeted energy-depletion attacks and supporting distinct consumption profiles for rechargeable and non-rechargeable IoT nodes. Section VI-A describes the experimental platform and parameter settings, Section VI-B examines the network under attack, and Section VI-C evaluates energy behavior in the absence of an adversary.

### 6.1. Experimental Setup

The proposed DEPRONN-ES framework was developed for the Contiki-NG operating system and assessed within the Renode simulation environment [35,36], which runs Contiki-based software while reproducing the behavior of resource-constrained IoT devices. The emulated hardware platform incorporated a 16-bit MSP430 Series 5 microcontroller equipped with 16 kB of RAM and 128 kB of flash storage, and network connectivity was modeled through a TI CC2520 radio transceiver operating in the 2.4 GHz band, compatible with IEEE 802.15.4 and 6LoWPAN.

Battery behavior for both replenishable and non-replenishable nodes was reproduced using the KiBaM, while the charging profiles of the sustainable nodes were derived from indoor illumination measurements collected at Columbia University [35,49]. The harvesting setup assumed a 170 cm^2^ photovoltaic panel operating with a 5 V input and 20% energy-conversion efficiency. KiBaM was coupled with Powertrace [50] to account for energy expenditure across radio transmission and reception, channel listening while idle, active processor execution, and low-power processor modes.

The principal system settings and experiment-specific parameters are consolidated in Table 2. Each attack and attack-free simulation covered a period of 72 h.

The two evaluations address different questions. The attack experiment considers 10 legitimate nodes and one malicious node to examine energy-assisted targeting and the effect of privacy perturbation. The attack-free evaluation considers 30 devices to examine energy behavior across heterogeneous network compositions. Because network size and attack conditions differ between these evaluations, their results should be interpreted separately; they do not establish the effect of increasing network size. These experiments constitute a small-scale proof of concept rather than a demonstration of scalability for smart-city deployments. Performance at larger scales, including the effects of contention, routing depth, control-message overhead, and forwarding workload, remains unverified. The 72-h duration captures three daily cycles of the supplied harvesting trace but does not establish long-term battery lifetime or seasonal harvesting performance.

The selected protocol stack was considered appropriate for the targeted smart-city monitoring applications, which do not impose stringent hard real-time latency requirements. The evaluation therefore focuses on attack effectiveness and energy behavior rather than end-to-end delay. Nevertheless, the absence of strict real-time requirements does not eliminate the need to assess delay and packet delivery when evaluating larger networks. These performance dimensions remain outside the reported evaluation.

### 6.2. Robustness to Energy-Depletion Attacks

The attack evaluation involved 10 benign IoT nodes and one adversarial node. By monitoring the exchanged EI, the adversary attempted to identify the node with the lowest residual battery level and direct legitimate-looking traffic toward it. Performance was assessed using three measures, which are detailed below: the Success Attack Rate (SAR), the battery consumption of the lowest-energy node, and average network-wide battery consumption.

#### 6.2.1. Attack-Targeting Accuracy

**Figure 6:**
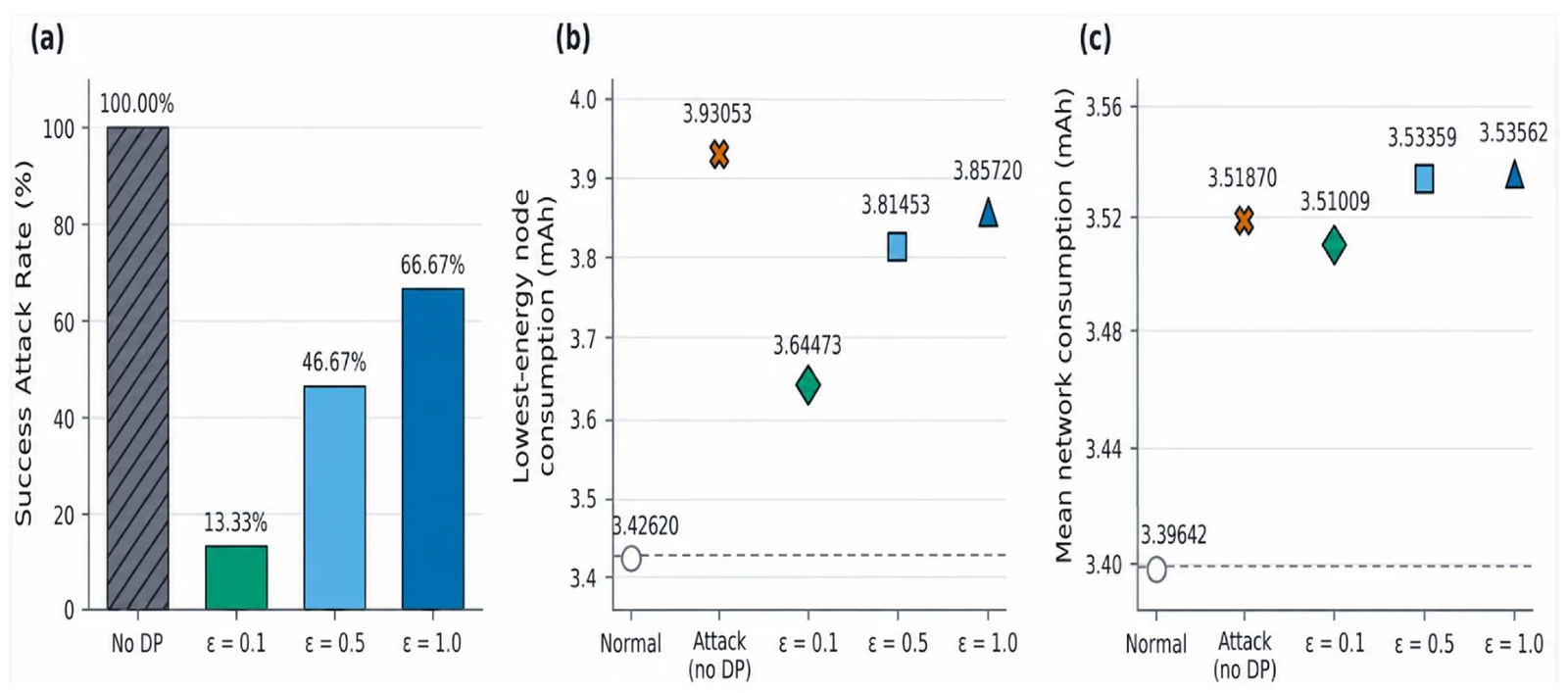
Attack performance under different privacy budgets: (**a**) Success Attack Rate, (**b**) battery consumption of the lowest-energy node, and (**c**) average network-wide battery consumption.

Figure 6a presents the SAR, defined as the proportion of attack attempts in which the adversary correctly identified the node with the lowest residual energy. Without differential privacy, the SAR reached 100%, indicating that unprotected EI enabled the adversary to identify the most vulnerable node in every attempt. However, the application of differential privacy substantially reduced the adversary’s targeting accuracy. At ε = 0.1, the SAR decreased to 13.33%, representing an 86.67-percentage-point reduction relative to the unprotected configuration. The SAR increased to 46.67% at ε = 0.5 and 66.67% at ε = 1.0, showing that stronger perturbation was associated with lower targeting accuracy in the evaluated conditions.

SAR measures successful identification of the lowest-energy node, not successful delivery or prevention of malicious traffic. Consequently, the reduction in SAR does not imply that attacks ceased or that malicious packets were blocked. When the adversary selects a different target, that node can still receive and process attack traffic, incurring additional energy consumption.

These results support reduced reliability of energy-assisted target identification under the tested conditions, rather than complete concealment of energy-constrained nodes. The evaluation also does not establish equivalent protection against persistent adversaries that combine successive announcements to improve their estimates.

#### 6.2.2. Energy Impact on the Targeted Node

Figure 6b reports the average battery consumption of the node with the lowest residual energy. Under normal operation without an attack, this node consumed 3.42620 mAh, and the unprotected attack increased its consumption to 3.93053 mAh. With ε = 0.1, consumption decreased to 3.64473 mAh, which was 0.28580 mAh lower than under the unprotected attack. The corresponding values were 3.81453 mAh at ε = 0.5 and 3.85720 mAh at ε = 1.0.

Despite the reduction at ε = 0.1, consumption remained 0.21853 mAh above the no-attack baseline. Consumption also remained above that baseline at ε = 0.5 and ε = 1.0. These results indicate partial mitigation of the energy impact on the lowest-energy node, rather than elimination of the attack. The privacy mechanism reduces the usefulness of disclosed EI for targeted selection; it does not directly detect, filter, or prevent malicious traffic.

#### 6.2.3. Network-Wide Energy Impact

As shown in Figure 6c, average network consumption increased from 3.39642 mAh under normal operation to 3.51870 mAh during the unprotected attack. The corresponding values under ε = 0.1, 0.5, and 1.0 were 3.51009, 3.53359, and 3.53562 mAh, respectively.

All three privacy configurations remained above the no-attack baseline. Consumption was slightly below the unprotected-attack value at ε = 0.1 and above it at ε = 0.5 and ε = 1.0. Thus, the reported averages do not demonstrate a consistent network-wide energy saving from privacy perturbation.

These findings are consistent with malicious traffic continuing to impose a communication and processing burden even when targeting becomes less accurate. The observed benefit is reduced accuracy in selecting the lowest-energy node, rather than removal of the attack burden across the network. A per-node consumption analysis would be required to determine how that burden is distributed among other nodes.

Together with Section 6.2.2, these results provide a partial assessment of the privacy–utility trade-off: lower targeting accuracy is accompanied by reduced consumption of the lowest-energy node under some privacy settings, but not by consistent network-wide savings. The measurements combine the effects of privacy perturbation, routing decisions, and attack behavior. They therefore do not separately quantify the computational cost of generating noise or the energy cost of routing with less accurate information.

The routing-utility cost also remains unquantified because packet delivery, end-to-end delay, control-message overhead, and parent-selection stability were not measured. A complete assessment requires matched comparisons with and without DP under both attack-free and attack conditions, keeping topology, offered traffic, harvesting conditions, and other parameters consistent. Such comparisons would distinguish the operational costs of privacy from their benefits against targeted attacks.

The continuing energy cost of attack traffic motivates combining EI privacy with complementary traffic-control mechanisms. Candidate defenses include per-source rate limiting, anomaly-based detection, and filtering of traffic identified as malicious. These mechanisms would aim to reduce accepted attack traffic, while privacy perturbation limits the information available for target selection. However, rate limits and filtering can also disrupt legitimate traffic, and detection introduces processing and communication costs. Their combined effectiveness and overhead have not been evaluated in the present study.

### 6.3. Energy Management Without an Attack

The attack-free evaluation examines how DEPRONN-ES manages heterogeneous energy resources in a partially sustainable IoT network. Residual battery energy is used to assess sustainable nodes because it reflects both harvested and consumed energy, while energy consumption is evaluated separately for traditional nodes because their batteries cannot be replenished. These metrics indicate how effectively DEPRONN-ES balances harvesting-aware operation with the conservation of non-rechargeable energy resources.

#### 6.3.1. Energy Scenarios and Network Composition

Low-residual-energy scenario. Each constrained node began with 50 mAh, corresponding to 5% of its battery capacity, and generated an average of 30 sensor records per minute. This scenario represents a resource-scarce operating condition with a moderate application workload.

High-residual-energy scenario. Each constrained node began with 850 mAh, corresponding to 85% of its battery capacity, and generated an average of 120 sensor records per minute. This scenario represents abundant stored energy combined with a substantially heavier application workload.

The initial battery levels of 5% and 85% provide contrasting energy-scarce and energy-abundant conditions. The associated traffic rates differ by a factor of four, introducing different communication workloads as well as different initial energy reserves. Consequently, these scenarios assess combined operating conditions rather than independently isolating the effects of battery charge and traffic intensity. Differences in consumption between the scenarios cannot be attributed solely to initial energy availability or the energy-management mechanism. Isolating these effects requires comparisons at a common traffic rate and, separately, at a common initial battery level.

The reported generation rates refer to raw sensor records rather than individual MQTT-SN packets. DEPRONN-ES aggregates these records into MQTT-SN QoS 0 PUBLISH messages, with each publication carrying up to 18 four-byte sensor records. The record-generation rate therefore represents the offered application workload; the resulting packet-transmission rate also depends on aggregation behavior.

**Figure 7:**
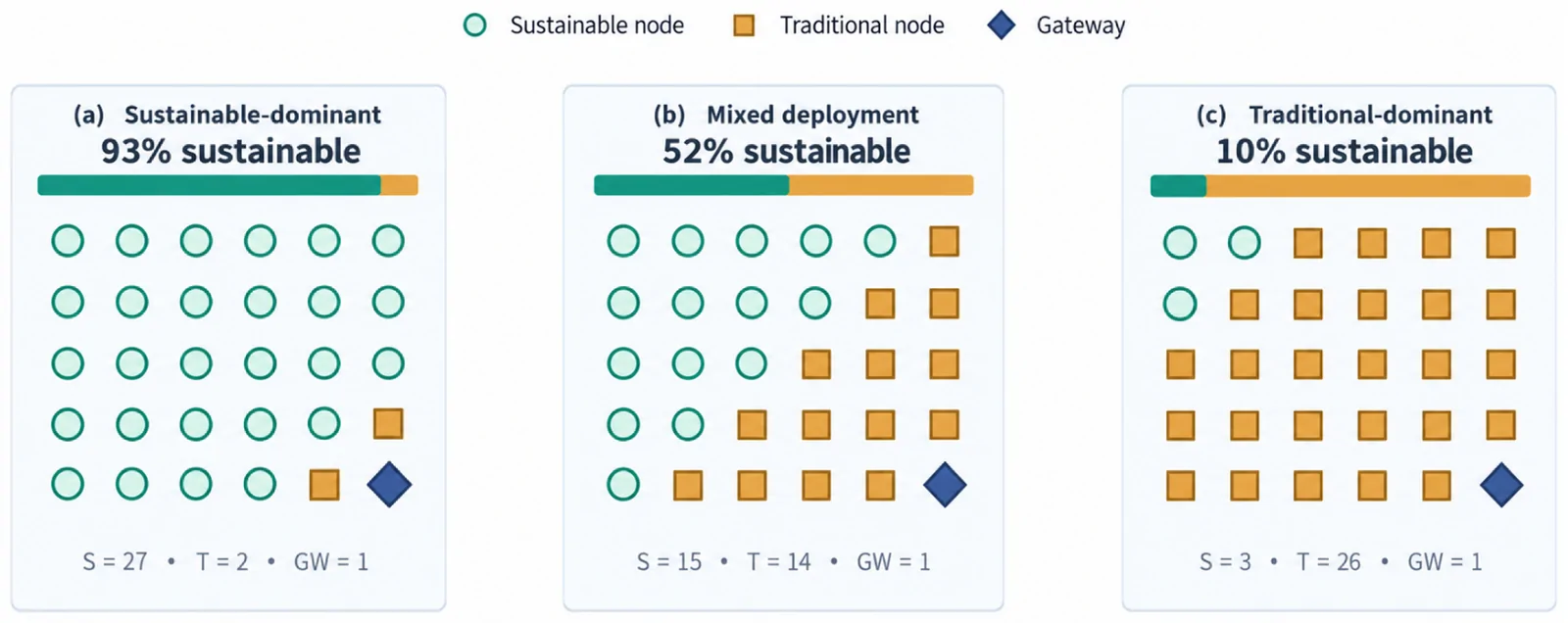
Illustrative sustainable IoT network compositions; sustainable-node percentages exclude the gateway.

The seven network configurations represented in Figure 7 each contain 30 devices: one externally powered gateway and 29 energy-constrained MQTT-SN clients. These configurations contain 27, 24, 20, 15, 10, 6, and 3 sustainable clients, paired with 2, 5, 9, 14, 19, 23, and 26 traditional clients, respectively. Sustainable-node proportions are calculated relative to the 29 clients, excluding the continuously powered gateway. Rounded to the nearest whole percentage, these proportions are 93%, 83%, 69%, 52%, 34%, 21%, and 10%.

These configurations span deployments dominated by sustainable nodes through deployments dominated by traditional nodes. They permit examination of energy behavior as the availability of harvesting-enabled devices changes at a fixed total node count. However, node placement and connectivity also affect the availability of sustainable forwarding paths; the sustainable-node proportion alone does not determine routing behavior. Because the total network size remains fixed, this comparison concerns network composition rather than scalability.

#### 6.3.2. Residual Energy Performance of Sustainable Nodes

**Figure 8:**
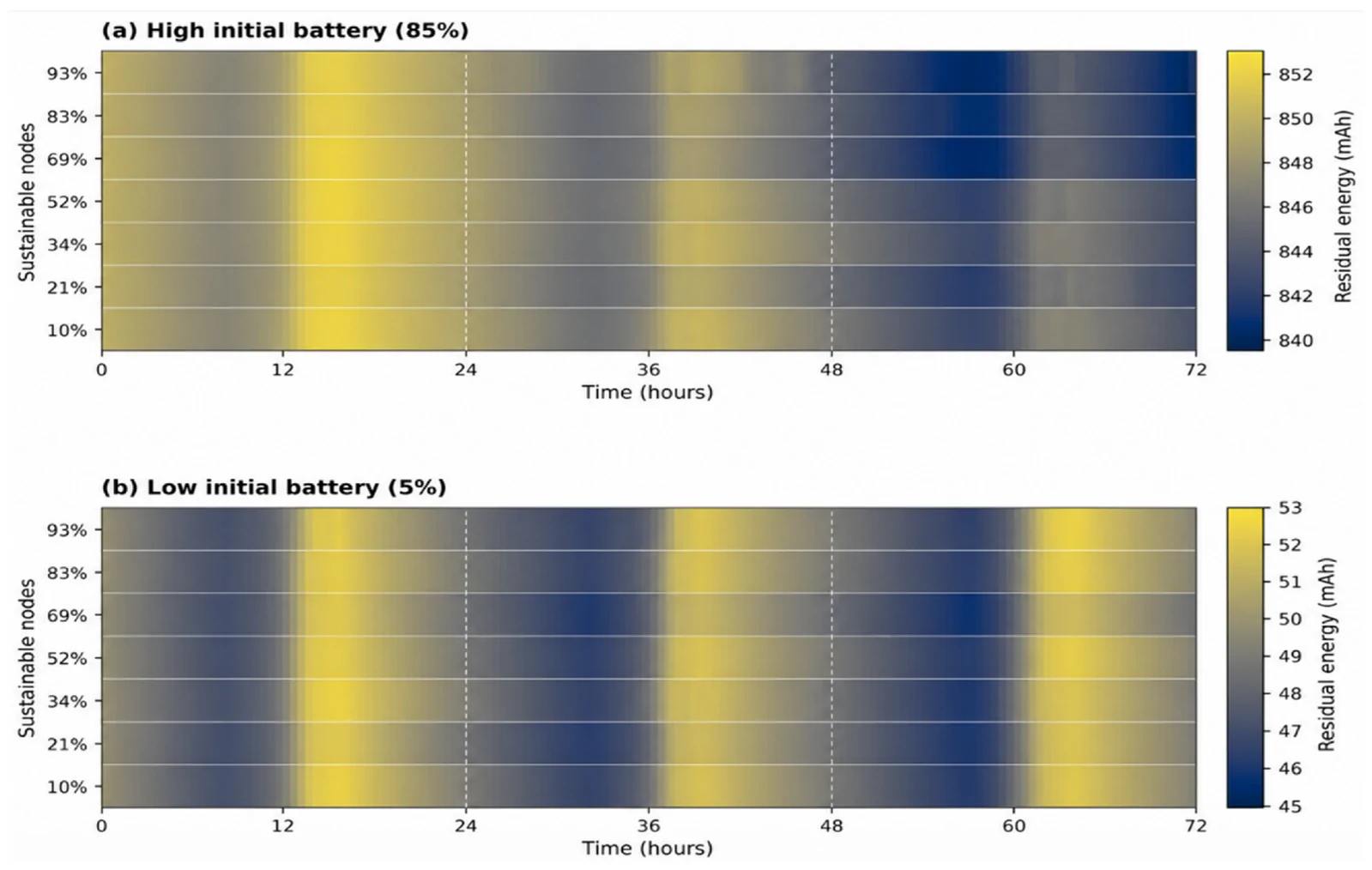
Residual energy of sustainable nodes over the 72-h simulation under (**a**) high (85%) and (**b**) low (5%) initial battery conditions.

Figure 8a presents the residual energy of sustainable nodes initialized at 85% of their battery capacity. All network compositions exhibited an overall decline across the three-day simulation, with recurring peaks and valleys generated by the solar-harvesting trace. The net decrease in residual energy is consistent with consumption exceeding harvested energy for the sustainable nodes represented in the figure. The trajectories also show that their residual-energy levels vary with network composition.

Figure 8b presents the results obtained when the batteries were initialized at 5% of their capacity. The residual-energy trajectories exhibit recurring increases during harvesting periods and decreases between them. These patterns illustrate the changing balance between harvested energy and consumption in sustainable nodes. However, sustainable-node residual energy alone does not establish whether harvesting exceeds aggregate consumption across the entire network, which also includes traditional nodes.

The seven configurations contain 27, 24, 20, 15, 10, 6, and 3 sustainable clients out of 29, respectively. Percentage labels exclude the gateway and are rounded to the nearest whole percentage.

#### 6.3.3. Energy Consumption of Traditional Nodes

**Figure 9:**
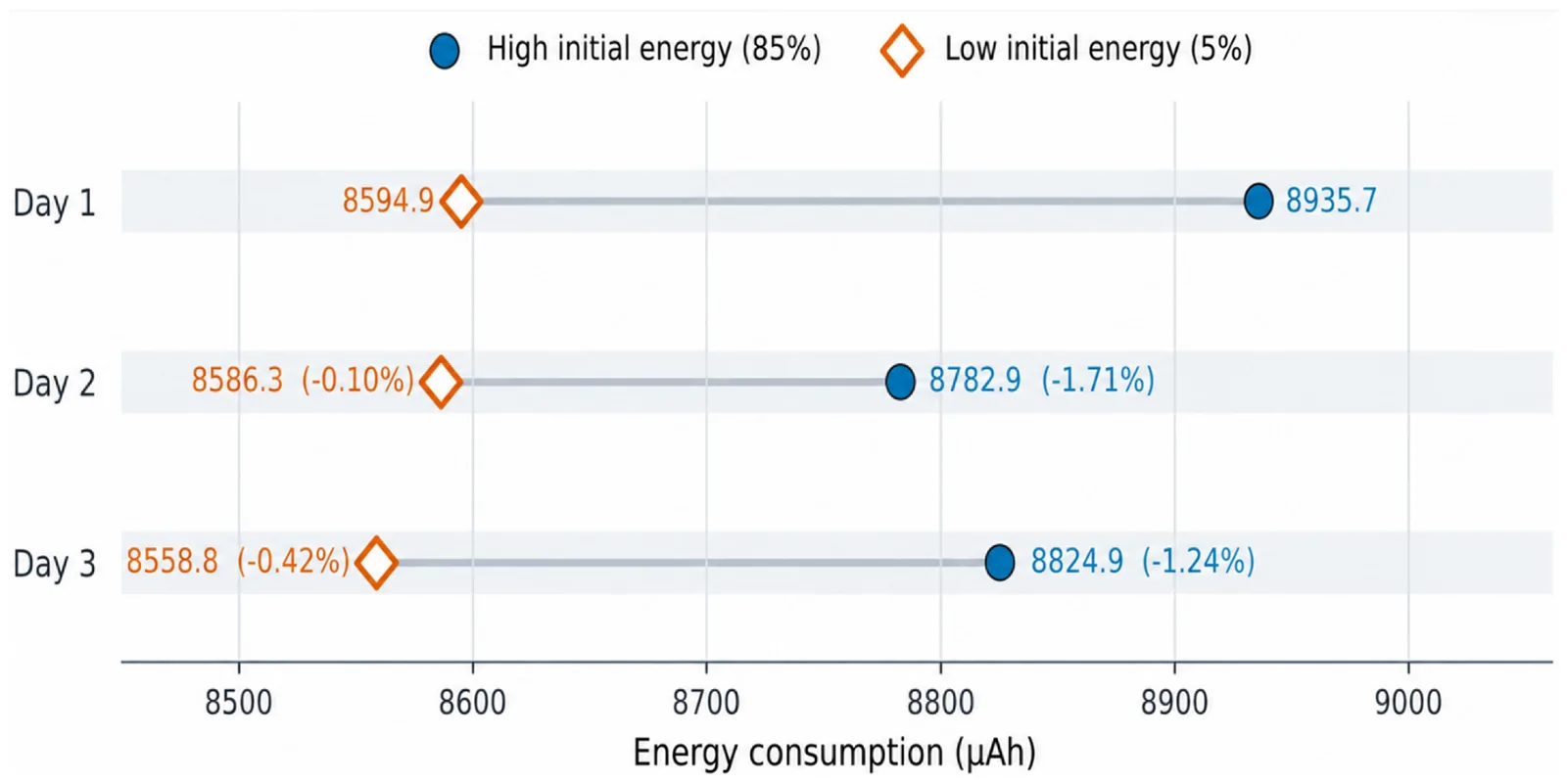
Relative changes in the energy consumption of traditional nodes on Days 2 and 3, using Day 1 as the baseline.

Figure 9 presents the energy consumed by traditional nodes on Day 1 and the relative changes observed on Days 2 and 3. Day-1 consumption was 8.9357 mAh under the high-initial-energy condition and 8.5949 mAh under the low-initial-energy condition. Thus, consumption in the low-energy scenario was 0.3408 mAh, or approximately 3.81%, lower than in the high-energy scenario. This difference indicates that DEPRONN-ES assigns fewer energy-intensive communication responsibilities when the available energy is limited.

Using Day 1 as the baseline, energy consumption under the 85% initial-battery condition decreased by 1.71% on Day 2 and 1.24% on Day 3. Under the 5% condition, the reductions were 0.10% and 0.42%, respectively. Therefore, consumption remained below the Day-1 level throughout both scenarios, although the magnitude of the reduction varied across days and initial-energy conditions.

DEPRONN-ES preserves the energy resources of traditional nodes while adapting routing and aggregation decisions to the prevailing network-energy state. The lower Day-1 consumption observed in the 5% scenario is also consistent with a more conservative operating profile under energy-scarce conditions.

## 7. Conclusions and Future Work

The transition toward fully sustainable smart-city infrastructure will inevitably involve heterogeneous IoT deployments in which rechargeable, energy-harvesting devices operate alongside legacy nodes powered by non-rechargeable batteries, devices that follow fundamentally different energy-management objectives. Conventional nodes must conserve their finite energy reserves to prolong operational lifetime, whereas energy-harvesting nodes can consume replenished energy while maintaining energy-neutral operation. However, exchanging EI for energy-aware routing may disclose the condition of individual batteries, allowing adversaries to identify weak devices and subject them to targeted energy-depletion attacks.

This study examines the existing DEPRONN-ES framework and its coordination of device-specific energy management with privacy-preserving EI dissemination. Energy-aware routing, data aggregation, differential privacy, RPL, and energy harvesting are established techniques; the focus here is their interaction within partially sustainable IoT networks. The original integration belongs to the prior DEPRONN-ES work discussed in Section 2.4 and is not claimed as a new contribution of this study. DEPRONN-ES selects forwarding parents and determines data-aggregation levels based on route-level energy availability and the power-source characteristics of participating devices. Before EI is transmitted to neighboring nodes, a differential-privacy mechanism adds controlled noise to the reported values. This coordination creates a trade-off: perturbation can reduce the accuracy of adversarial energy estimates while also affecting the information used for legitimate routing and aggregation decisions.

The reported evaluation suggests security and energy-management benefits under the tested conditions. Without privacy protection, the attacker identified the lowest-energy node in 100% of its attempts. At ε = 0.1, SAR decreased to 13.33%, an 86.67-percentage-point reduction, and the targeted node consumed 0.28580 mAh less energy than under the unprotected attack. However, its consumption remained above the no-attack baseline. These results concern reduced targeting accuracy and attack impact, rather than prevention of malicious traffic. In the attack-free experiments, traditional-node consumption on Day 1 was 0.3408 mAh lower in the low-energy scenario than in the high-energy scenario, while the residual-energy trajectories of sustainable nodes followed the available harvesting conditions. Because the low- and high-energy scenarios also used different traffic-generation rates, their consumption difference cannot be attributed solely to the initial battery level.

The evaluation nevertheless has scalability and external-validity limitations. The reported attack setting contained 10 legitimate nodes and one malicious node, while the attack-free setting contained 30 devices. Because network size and attack conditions differ between these evaluations, their comparison does not isolate the effect of network size. Similarly, changing initial battery charge and traffic intensity together prevents their individual effects from being established. The experiments also used a 72-h horizon, a single indoor-light harvesting dataset, and fixed privacy budgets. These settings do not establish long-term network lifetime or robustness across different harvesting conditions. The findings should therefore be interpreted within this trace-driven and small-scale configuration. The available comparisons do not isolate the contribution of every component or establish superiority over alternative integrated approaches. The energy measurements also do not isolate the computational overhead of DP from changes in routing and attack behavior. Packet delivery, end-to-end delay, control-message overhead, and parent-selection stability were not quantified. Consequently, the evaluation does not establish the routing-utility cost of privacy perturbation or an optimal privacy-budget setting. The evaluation does not establish resistance to persistent adversaries that combine repeated DIO announcements over time. Temporal averaging may improve energy estimates, particularly when the underlying values change slowly, and per-announcement privacy budgets do not constitute an equivalent privacy guarantee for the complete observation period.

Further evaluation should include matched baseline comparisons and component-level ablations to distinguish the effects of dual energy profiling, aggregation, and privacy perturbation. Controlled experiments should vary initial battery charge and traffic intensity independently and compare network sizes under matched attack and workload conditions. Repeated runs with documented seeds and uncertainty estimates are needed to assess the stability of the results. Future work should assess DEPRONN-ES on larger physical IoT testbeds, such as FIT IoT-LAB, and investigate reinforcement-learning methods at the edge for more granular and adaptive energy profiling. Privacy research should compare differential privacy with complementary anonymization techniques while distinguishing their respective threat models and guarantees. Additional experiments should examine heterogeneous harvesting sources, longer observation periods, adaptive privacy budgets, and broader attack configurations. Per-node energy distributions, predefined network-lifetime endpoints, packet delivery, delay, and routing overhead should be measured to characterize both benefits and operational costs. Future work should also evaluate temporal-averaging attacks and investigate privacy accounting across repeated releases, total-budget allocation, and continual-observation mechanisms.

## Figures and Tables

**Table 1:** Comparison of privacy, energy-management, and routing approaches for smart-city IoT networks.

Study	Heterogeneous Energy Sources	Energy-Aware Routing	Data Aggregation	EI Privacy	Explicit Attack Model	Constrained-IoT Evaluation
Hossain et al. [22]	–	–	–	✔	–	–
Pan et al. [23]	–	–	–	✔	✔	–
Nandhini et al. [24]	–	✔	–	–	✔	✔
Ni et al. [25]	–	◐	–	–	–	–
Nguyen et al. [26]	–	✔	–	–	–	–
Said et al. [27]	✔	–	✔	–	–	–
Sadek et al. [28]	✔	◐	–	–	–	–
Sharanya et al. [29]	◐	–	–	–	–	–
Bathre et al. [30]	◐	–	–	–	–	–
DEPRONN-ES	✔	✔	✔	✔	✔	✔

Note: ✔ = explicitly addressed; ◐ = partially addressed or addressed at the device level rather than across heterogeneous network nodes; – = not addressed.

**Table 2:** Evaluation parameters.

Parameter	Value
**Common configuration**	
Simulator	Renode
IoT operating system	Contiki-NG 3.0
Simulated hardware	MSP430 Series 5
Protocol stack	IEEE 802.15.4, 6LoWPAN, RPL, UDP, and MQTT-SN
Battery model	KiBaM
Energy-harvesting trace	Columbia University indoor-light trace
Total battery capacity	1000 mAh
Energy-broadcast interval	Trickle algorithm: minimum 125 ms; maximum 109 min
**Evaluation 1: Network under energy-oriented attack**	
Legitimate nodes	10
Malicious nodes	1
Simulation duration	72 h
Privacy settings	No privacy; ε = 0.1; ε = 0.5; ε = 1.0
**Evaluation 2: Network without an attack**	
Total nodes	30
Rechargeable nodes	30, 28, 25, 21, 16, 11, 7, and 4
Non-rechargeable nodes	0, 2, 5, 9, 14, 19, 23, and 26
Malicious nodes	0
Simulation duration	72 h
Low residual energy	50 mAh
Traffic under low residual energy	30 MQTT-SN messages per minute
High residual energy	850 mAh
Traffic under high residual energy	120 MQTT-SN messages per minute

## Data Availability

Data can be made available upon request, to ensure that privacy restrictions are upheld.
